# Design of Experiment Approach to Modeling the Effects of Formulation and Drug Loading on the Structure and Properties of Therapeutic Nanogels

**DOI:** 10.1021/acs.molpharmaceut.1c00699

**Published:** 2022-01-21

**Authors:** Hei Ming Kenneth Ho, Duncan Q. M. Craig, Richard M. Day

**Affiliations:** † School of Pharmacy, University College London, 29-39 Brunswick Square, London WC1N 1AX, U.K.; ‡ Centre for Precision Healthcare, UCL Division of Medicine, University College London, 5 University Street, London WC1E 6JF, U.K.

**Keywords:** nanogels, tunable, design of experiment, chitosan, prediction

## Abstract

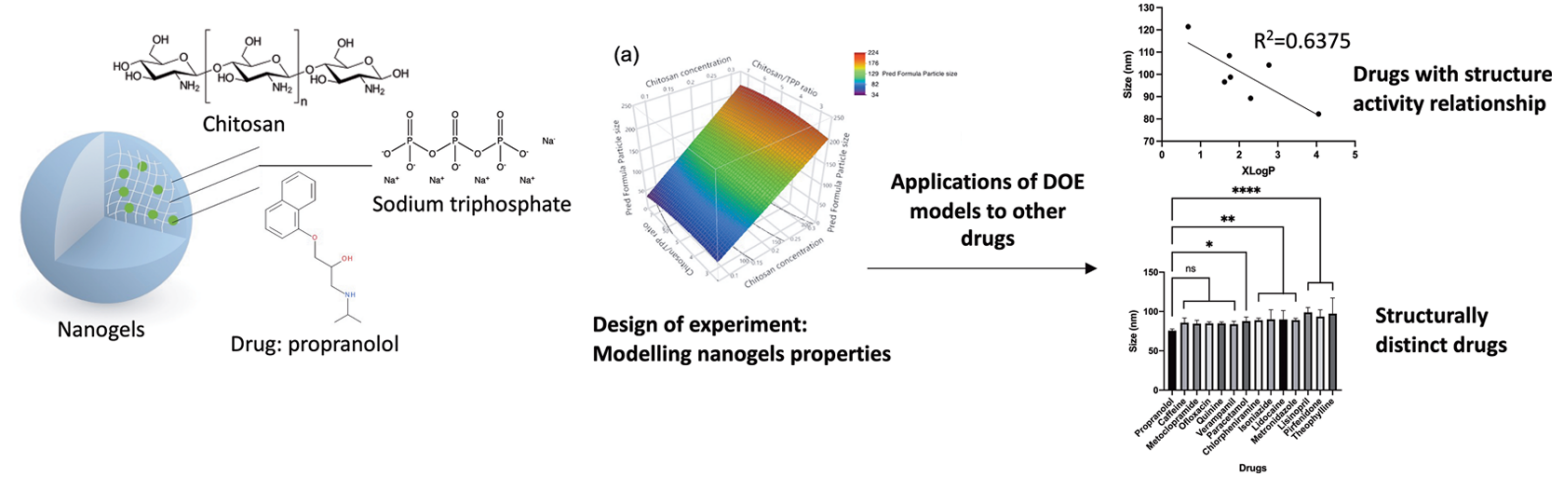

The physical properties of nanoparticles may affect the uptake mechanism, biodistribution, stability, and other physicochemical properties of drug delivery systems. This study aimed to first develop a model exploring the factors controlling the nanogel physical properties using a single drug (propranolol), followed by an evaluation of whether these models can be applied more generally to a range of drugs. Size, polydispersity, ζ potential, and encapsulation efficiency were investigated using a design of experiment (DOE) approach to optimize formulations by systematically identifying the effects of, and interactions between, parameters associated with nanogel formulation and drug loading. Three formulation factors were selected, namely, chitosan concentration, the ratio between the chitosan and cross-linker—sodium triphosphate—and the ratio between the chitosan and drug. The results indicate that the DOE approach can be used not only to model but also to predict the size and polydispersity index (PDI). To explore the application of these prediction models with other drugs and to identify the relationship between the drug structure and nanogel properties, nanogels loaded with 12 structurally distinct drugs and 6 structurally similar drugs were fabricated at the optimal condition for propranolol in the model. The measured size, PDI, and ζ potential of the nanogels could not be modeled using distinct DOE parameters for dissimilar drugs, indicating that each drug requires a separate analysis. Nevertheless, for drugs with structural similarities, various linear and nonlinear trends were observed in the size, PDI, and ζ potential of nanogels against selected molecular descriptors, indicating that there are indeed relationships between the drug molecular structure and the performance outcomes, which may be modeled and predicted using the DOE approach. In conclusion, the study demonstrates that DOE models can be applied to model and predict the influence of formulation and drug loading on key performance parameters. While distinct models are required for structurally unrelated drugs, it was possible to establish correlations for the drug series investigated, which were based on polarity, hydrophobicity, and polarizability, thereby elucidating the importance of the interactions between the drug and the nanogels based on the nanogel properties and thus deepening the understanding of the drug-loading mechanisms in nanogels.

## 1 Introduction

Nanogels, also known as hydrogel nanoparticles, are nanosized particles comprised of a network of hydrophilic polymers (hydrogel). They have a three-dimensional (3D) hydrophilic structure containing voids that allow the encapsulation of active pharmaceutical ingredients, ranging from small-molecule drugs to macromolecules such as peptides, proteins, and genes. They are formed by cross-linking hydrophilic natural and/or synthetic polymers either physically or chemically and possess strong water-holding ability without self-dissolution or self-disintegration. Beneficial uses of nanogels include protecting the drug cargo from metabolism, targeted delivery via enhanced permeation and retention (EPR) effects, or active targeting with the ligands conjugated on the polymer. This makes them ideal for delivering fragile therapeutics, such as proteins, enzymes, and genes. Chitosan and sodium triphosphate pentabasic (TPP) nanogels are the most extensively explored systems, which only require mild fabrication conditions to form nanogels via ionic gelation. Chitosan is a linear polycationic polysaccharide derived from chitin found in the cell wall of fungi, exoskeletons of arthropods, and shells in crustaceans.^1−3^ As it is a biodegradable and biocompatible, nontoxic, and mucoadhesive material,^4^ it is suitable for drug delivery.

Controlling the parameters of nanoparticles is crucial for any nanoparticle formulation, including nanogels. Size and surface charge of nanoparticles are especially important as these parameters impact the solubility, biodistribution, stability, cytotoxicity, cellular uptakes, and clearance of the nanoparticles. For instance, the uptake of nanoparticles into cells is dependent on the size, shape, surface hydrophobicity, and charge of the nanoparticles, although the permeability varies between different cells.^5^ Furthermore, Tang et al. showed that only cationic nanoparticles suspended in culture medium were ingested by endocytosis, suggesting the importance of nanoparticle surface charge in cellular uptake.^6^ Stability is another key parameter for nanoparticle formulation. In chitosan–TPP nanogels, the nanoparticles are stabilized by electrostatic repulsion and ζ potential (ZP) is an indication of the potential stability of the colloidal system. Particles with ζ potentials more positive than 30 mV or more negative than −30 mV are considered stable, and the nanoparticles are unlikely to flocculate due to the repulsion between nanoparticles.^7^ In short, the successful delivery of nanogels requires optimal size and charge, and thus, the fabrication process needs to be optimized.

Design of experiment (DOE) is a popular method for optimizing pharmaceutical formulation development. It allows a systematic evaluation of the effect of multiple factors in the variation of the response measured and reduces the number of experimental runs. However, the drawback is that the process is driven by experiments and is labor-intensive, as DOE models are constructed based on the input parameters, and any changes in the input factors require the construction of a new model. Thus, a model is usually reported for a particular drug and delivery system. The prediction ability of these models with new drug payloads is rarely explored in the literature and therefore is poorly understood. Indeed, the drugs are commonly thought to be encapsulated in the voids between polymer chains and thus the drug choice is often assumed to be of limited influence if that drug is of a size and solubility to allow incorporation into the nanogel voids. Here, we challenge this view by exploring both the development of predictive models using a DOE approach and the possible generalizability of both the approach and the model to a range of drugs. We suggest that a predictive understanding of the effects of composition on nanogel performance parameters will both aid the formulator and aid in understanding the role of drug structure on both incorporation and nanogel architecture.

The mechanism of drug loading in chitosan–TPP nanogels is not well understood. It is thought that the drugs are loaded in the voids formed between the cross-linked chitosan matrix during the gelation process. If the loading mechanism is purely entrapment during the gelation process, the nanogel properties should be governed by the formulation, such as the polymer and cross-linker concentrations. Thus, different drugs could be loaded into the nanogel without alteration of the nanogel properties, as long as the drugs fit into the voids of the nanogels and the prediction models remain valid for different drugs. Since these nanogels are fabricated via electrostatic interactions between chitosan and TPP, it is expected that the drug molecules could also potentially impact the gelation process, altering the interaction between the drug, chitosan, and TPP. Thus, the structure of drugs, drug properties, and pH are important. We hypothesize that drugs are loaded both via physical entrapment and through interaction with the nanogel carrier during the gelation process, where the drug electrostatically interacts with either oppositely charged chitosan or TPP. Other interactions such as weak van der Waal’s force and hydrophobic interaction could also play a role in the interactions. Thus, it is expected that the properties vary when the properties of the drug change.

To verify this hypothesis, we initially used propranolol as a model drug for nanogel fabrication. Response surface models were constructed for the unloaded and drug-loaded systems to predict the properties of propranolol-loaded chitosan/TPP nanogels, namely, hydrodynamic size, polydispersity index (PDI), ζ potential (ZP), and encapsulation efficiency (%EE). Seventeen experimental runs were performed to build a three-factor, three-level face-centered cubic (FCC) central composite model in which chitosan, chitosan/TPP ratio, and chitosan/propranolol ratio were varied. The models were then verified by an individual test group before identifying the optimal conditions. The optimal condition was selected and explored experimentally with 12 other drugs without structural similarity to propranolol and 6 β-blocker drugs with close structural relationships to propranolol, of which the latter are expected to similarly interact with the polymer or cross-linker. In this way, we intend to establish whether the models may be applied to other drugs and if so whether structural similarity is a requirement for such extrapolation.

To describe a drug molecule, one of the common representations is the molecular descriptor. It is defined as an algorithm-generated mathematical representation of structural or physicochemical properties of molecules,^8^ which can be classified based on either the dimensionality or information content. The former classifies descriptors from zero-dimensional (0D) to seven-dimensional (7D) descriptors, whereas the latter classifies them into constitutional, topological, geometric, and electronic descriptors.^9^ The classifications overlap extensively and are not mutually exclusive. Thus, molecular descriptors discussed in this study will be based on the constitutional and topological properties for clarity. Constitutional descriptors are calculated from molecular formulae, such as molecular weight atom and bond count, whereas topological and structural descriptors, including counts of fragments and functional groups, are calculated from the two-dimensional (2D) structure. Geometric spatial and electronic descriptors are derived from the three-dimensional (3D) structure.^9^ Numerous open-source and commercial software exist for computing these molecular descriptors, including PaDEL,^10^ MORDRED,^11^ CDK,^12^ Dragon,^13^ and RDKit.^14^ As a proof-of-concept study, these drugs were described in terms of 15 basic molecular descriptors, which include the number of acid (nAcid), base (nBase), rings (nRings), hydrogen bond acceptors (nHBAcc), hydrogen bond donors (nHBDon), the sum of the atomic polarizability (apol), sum of the absolute value of the difference between atomic polarizability of all bonded atoms (bpol) in the molecule, Wiener path number (WPATH), Wiener polarity number (WPOL), topological polar surface area (TopoPSA), topological diameter (TopoDiameter), Petitjean topological shape index (Toposhape), and two logarithms of *n*-octanol/water partition coefficients ALog *P* and XLog *P*. The constituent descriptors, especially the number of chemical groups, were selected to evaluate the chemical groups responsible for the interactions with the carriers and to identify the mode of loading in the nanogels, where the polarity and polarizability were picked to evaluate the effects of other interactions, such as van der Waal’s force and hydrophobic interaction. The topological shape and size descriptors were selected to evaluate whether the size and shape of the drugs impact drug entrapment since the drugs are loaded into the voids of the nanogels. The relationship between nanogel properties and molecular descriptors of drugs was determined via multiple linear regressions, logarithmic, exponential, and quadratics correlations. This facilitates our understanding of how a drug may influence entrapment and nanogel structure, with the intention of establishing the generalizability of the DOE modeling approach across a wide range of drug structures and increasing our understanding of the mechanism of drug loading in nanogels. Notably, the development of predictive models for optimizing the properties of drug delivery systems is a highly useful and well-established approach within the field. In this particular case, we focus on two as yet unexplored applications of performance modeling. First, the formulation of nanogels is a complex and as yet poorly predictable process whereby each system is explored on a largely individual basis due to the absence of a validated methodology for performance prediction; hence, there is a clear requirement and novel application for such approaches for these systems. Second, the effect of drug incorporation on performance and properties is a highly important area, which has as yet again not received systematic study; hence, our intention is to develop methodologies whereby the effects of incorporation of the active agent may be developed at least, in the present case, for structurally related molecules, with the intention of this providing a basis for studies into a broader range of active pharmaceutical ingredients.

## 2 Materials and Methods

### 2.1 Materials

Low-molecular-weight (LMW) chitosan was purchased from Sigma-Aldrich (St. Louis, MO) with a molecular weight of 50–190 kDa according to the manufacturer. Paracetamol, metoclopramide hydrochloride, metoprolol tartrate, lidocaine hydrochloride, theophylline, ofloxacin, metronidazole, acebutolol, pindolol, and esmolol hydrochloride were also acquired from Sigma-Aldrich (St. Louis, MO). Pentabasic sodium triphosphate (TPP) and caffeine were purchased from Fluka (Switzerland), while propranolol hydrochloride (Propranolol HCl), quinine anhydrous, lisinopril dihydrate, verapamil hydrochloride, and betaxolol were acquired from Acros Organics (Geel, Belgium). Pirfenidone and atenolol were purchased from Tokyo Chemical Industry (Tokyo, Japan). Chlorpheniramine maleate was acquired from the LKT Laboratory (St. Paul, MN). Glacial acetic acid was obtained from Fisher Scientific (Waltham, MA). Sodium hydroxide pellets were acquired from VWR (Radnor, PA). All chemicals were of analytic grade and used as supplied.

### 2.2 Propranolol-Loaded Nanogel Fabrication

Propranolol-loaded chitosan nanogels were prepared by the ionic cross-linking method, adapted from the method reported by Al-Kassas et al.^15^ Low-molecular-weight chitosan was first dissolved in 1% acetic acid solution until it formed a clear solution, followed by adjustment of the pH to 4.5 with 0.1 M sodium hydroxide solution. Chitosan solution was filtered through a 0.22 μm syringe filter before use. Propranolol HCl was weighed and dissolved in the chitosan solution before the addition of the TPP solution. Meanwhile, various amounts of TPP were dissolved in deionized water to prepare different concentrations and the TPP solutions were also filtered with a 0.22 μm syringe filter. An equal amount of TPP solution was added to the chitosan solution under stirring at room temperature. The solution was then stirred at 600 rpm for 1 h. A range of nanogels were prepared by varying these factors according to the experimental matrix shown in Table 1. The prepared nanogels were then kept in a fridge at 4 °C for further characterizations. All nanogels were prepared and tested in triplicate.

**Table 1:** Independent and Dependent Variables and the Experimental Design Matrix of Central Composite Design (CCD) Design

	independent variables			dependent variables			
sample	CC	CT	CP	size (nm)	ζ potential (mV)	PDI	encapsulation efficiency (%)
1	0.2	3	0.375	135.3	23.45	0.243	23.3
2	0.2	5	0.25	135.6	29.53	0.367	40.6
3	0.2	5	0.375	132.3	28.01	0.378	11.7
4	0.2	5	0.375	138.6	29.95	0.352	18.6
5	0.1	5	0.375	65.6	24.57	0.288	30.7
6	0.1	3	0.5	69.8	25.73	0.233	35.1
7	0.3	5	0.375	208.1	26.49	0.481	18.7
8	0.2	5	0.375	135.2	30.59	0.327	16.5
9	0.2	5	0.5	146.4	29.62	0.323	20.9
10	0.1	7	0.25	59.4	18.67	0.342	19.7
11	0.3	3	0.25	194.4	24.38	0.343	21.7
12	0.3	7	0.25	198.6	31.58	0.498	14.5
13	0.3	7	0.5	206.2	31.02	0.507	16.4
14	0.2	7	0.375	132.0	32.23	0.461	18.0
15	0.1	3	0.25	65.0	18.11	0.217	26.7
16	0.3	3	0.5	190.8	24.69	0.345	19.6
17	0.1	7	0.5	56.8	25.25	0.313	25.8

### 2.3 Experimental Design

Response surface methodology was used to determine the optimal condition for preparing propranolol-loaded nanogels. A face-centered cubic (FCC) central composite design (CCD) was used in the optimization, which was formed by three factors, namely, the chitosan concentration (CC), the chitosan–TPP mass ratio (C/T), and the chitosan–propranolol mass ratio (C/P), at three levels, as shown in Table S1. A total of 17 experimental runs were denoted as the training set and were performed in triplicate to construct the response surface model. The composite matrix was constructed using JMP 15 (SAS Institute, Cary, NC). Four properties of nanogels (*Z*-average (size), ZP, PDI, and %EE), which contribute to being a successful drug carrier, were determined as the dependent variables.

A stepwise least-squares regression was used to fit the polynomial model to the data individually for each dependent variable. Fivefold cross-validation was performed to validate the model for all dependent variables. One-way analysis of variation (ANOVA) test and lack-of-fit test were conducted to determine the statistical significance and goodness of fit for the model, respectively, at a confidence interval (CI) of 95%. Response surfaces were plotted to visualize the relationship between independent and dependent variables. A *p*-value <0.05 is considered statistically significant.

#### 2.3.1 Multiple Response Optimization (MRO)

Multiple response optimization was employed to determine the optimal condition for fabricating propranolol-loaded nanogels, as the dependent variables might contradict each other. The desirability function approach, first proposed by Harrington^16^ in 1965 and later advocated by Derringer and Suich,^17^ is one of the most widely used methods in multiple response optimization. It transformed the response variables (*y*_*i*_) into an individual desirability function *d*_*i*_(*y*_*i*_), with a number assigned between 0 and 1. *d*_*i*_(*y*_*i*_) = 0 indicates a completely undesirable response, while *d*_*i*_(*y*_*i*_) = 1 represents the most desirable response. Individual desirability functions were transformed using JMP 15 software to minimize the particle size and PDI while maximizing the %EE and ZP. Individual desirability functions were then combined into overall desirability, as shown in eq 1
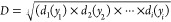
1where *d*_1_(*y*_1_) and *d*_2_(*y*_2_) denote the individual desirability function for factors 1 and 2, respectively, *i* is the total number of factors, and *d*_*i*_(*y*_*i*_) is the individual desirability function of factor *i*.

The running conditions with the highest overall desirability were deemed as the optimal condition and were determined by JMP 15. Nanogels were then fabricated under the optimal conditions in triplicate, with the dependent variables measured experimentally and compared with the predicted values to validate the models. Nanogels produced were then freeze-dried and characterized.

#### 2.3.2 Test Set Validation and Final Formulations

To determine the predictive accuracy of the models, an independent test set with 13 formulations was used, with the properties of nanogels measured experimentally and compared with the predicted value from the model. The experimental conditions for the formulations in the training set were generated randomly, and the test set is reported in Table 2. Finally, nanogels were also fabricated under experimental conditions predicted by the validated models to obtain 100 and 200 nm in size, with the secondary aim to minimize the PDI. Owing to the low predictability of the ZP and %EE models, these parameters were measured but not compared with the predicted values.

**Table 2:** Parameter Investigated, Experimental Findings, and Predicted Results of the Test Set for Evaluating the Predictive Accuracy of the CCD Models

	experiment conditions			size (nm)			PDI			ZP (mV)			EE (%)		
	CC	C/T	C/P	exp	pred	%diff	exp	pred	%diff	exp	pred	%diff	exp	pred	%diff
1	0.15	4	0.25	114.4	103.0	–10.0	0.268	0.281	4.9	27.4	23.8	4.9	14.9	26.2	75.8
2	0.25	4	0.375	138.1	168.8	22.2	0.283	0.353	24.7	25.4	27.8	24.7	15.4	21.5	39.6
3	0.25	6	0.25	131.1	170.9	30.4	0.318	0.434	36.5	25.1	30.4	36.5	9.5	18.3	92.6
4	0.1	4	0.5	98.9	70.1	–29.1	0.156	0.246	57.7	29.5	25.4	57.7	19.6	28.6	45.9
5	0.15	3	0.375	118.1	99.4	–15.8	0.245	0.248	1.2	26.0	25.4	1.2	15.4	27.8	80.5
6	0.3	6	0.5	179.8	206.2	14.7	0.422	0.476	12.8	29.4	29.2	12.8	8.5	16.0	88.2
7	0.15	3	0.25	119.3	99.4	–16.7	0.253	0.248	–2.0	26.3	23.1	–2.0	31.4	27.8	–11.5
8	0.2	4	0.5	113.0	135.9	20.3	0.268	0.317	18.3	23.1	29.3	18.3	9.7	23.9	146.4
9	0.2	6	0.375	107.6	135.6	26.0	0.289	0.391	35.3	24.0	30.2	35.3	25.2	20.7	–17.9
10	0.1	6	0.5	60.7	65.1	7.2	0.146	0.306	109.6	24.8	26.0	109.6	18.5	25.4	37.3
11	0.15	7	0.375	115.8	94.1	–18.7	0.355	0.382	7.6	26.1	28.2	7.6	13.8	21.4	55.1
12	0.25	3	0.25	133.8	162.7	21.6	0.263	0.312	18.6	25.0	25.8	18.6	18.3	23.1	26.2
13	0.3	4	0.5	194.4	201.6	3.7	0.397	0.388	–2.3	28.4	25.3	–2.3	18.8	19.2	2.1

### 2.4 Characterization Techniques for Raw Materials and Freeze-Dried Nanogels

#### 2.4.1 Fourier Transform Infrared (FTIR) Spectroscopy

Analysis was performed with a Spectrum 100 FTIR spectrometer equipped with an attenuated total reflectance (ATR) sampling accessory (PerkinElmer, Waltham) in the range of 650–4000 cm^–1^ and with a resolution of 1 cm^–1^.

### 2.5 Characterization Techniques for Nanogels

#### 2.5.1 Transmission Electron Microscopy

The shape and morphology of the nanogels were characterized by an FEI CM120 Bio Twin Transmission Electron Microscope (TEM) (Hillsboro, OR). One drop of the nanogel sample was dropped onto 200-mesh carbon lacey-coated copper grids and stained with 1% uranyl acetate solution, followed by air-drying at room temperature for a few minutes. The excess solution was removed using filter paper. Particle size distribution was performed using ImageJ (NIH, Bethesda, MA).

#### 2.5.2 Dynamic Light Scattering (DLS) and Electrophoretic Light Scattering

The average diameter and polydispersity of the nanogels were measured using a Zetasizer Ultra (Malvern Panalyticals, Malvern, U.K.) at room temperature using a backscatter angle of 173°. A disposable polystyrene cuvette was employed in the analysis. ζ potentials were measured using U-shaped capillary cells (DTS 1070, Malvern Panalytical, Malvern, U.K.). The results were measured in triplicate obtained from three independent experiments.

#### 2.5.3 Encapsulation Efficiency of Propranolol in Chitosan/TPP Nanogels

Measurement of %EE of propranolol was adapted from the method reported by Al-Kassas et al.^15^ Instead of separating the nanogels via centrifugation solely, 0.5 mL of the propranolol-loaded nanogel solutions were loaded into a 0.5 mL Amicon diafiltration tube (molecular weight cut-off (MWCO) 3000; Merck Millipore, Billerica, MA). The solutions were then centrifuged at 14 000*g* for 30 min at 4 °C using a refrigerated mini centrifuge (Heraeus Fresco 17, Thermo Scientific, Waltham), and the filtrate was isolated and assayed by a UV–vis spectrometer (Jenway 6305, Vernon Hills, IL) at a wavelength of 280 nm. A range of concentrations between 5 and 100 μg/mL were prepared to construct the calibration curve, which is shown in Figure S1. %EE was calculated using eq 2. The experiment was repeated three times, and the results were presented as mean ± standard deviation (SD)
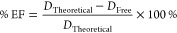
2where *D*_Theoretical_ refers to the amount of propranolol added into the solution, while *D*_Free_ refers to the amount of propranolol present in the aliquot after centrifugation.

### 2.6 Drug Release of Propranolol Loaded in Chitosan/TPP Nanogels

Dissolution tests were performed in 50 mL phosphate-buffered saline (PBS) (10 mM, pH 7.4) solution with continuous stirring at 37 °C for 72 h. Two milliliters of the nanogel solutions were loaded into a cellulose dialysis bag (3500 MWCO, volume/cm = 1.91, Fischer Scientific, Waltham, MA) with both ends tied, followed by submerging into PBS. One milliliter of aliquot was withdrawn at certain time points, and an equal volume of the fresh preheated PBS solution was added to maintain a constant volume. Propranolol was assayed by UV–vis spectroscopy using a UV–vis spectrometer (Jenway 6305, Vernon Hills, IL). The wavelength was set at 280 nm, and drug concentrations were calculated using predetermined calibration curves. The experiment was replicated independently three times, and the results were presented as the mean value ± standard derivation.

### 2.7 Application of the Validated Model with Other Drugs

To determine the possibility of applying the validated models to other drugs, nanogels were fabricated at the optimal condition, as discussed in Section 2.3.2. Chitosan nanogels loaded with other drugs were prepared by the same method discussed, except for atenolol. Atenolol-loaded chitosan nanogels were prepared with the respective amount of atenolol dissolving in the chitosan before pH adjustment, as the atenolol is not in a salt form, of which the pH of the chitosan solution would increase upon addition after pH adjustment. Drugs with and without structural relationships were grouped and analyzed separately.

Owing to the poor predictability of the models, ζ potentials of the nanogels were measured but not predicted. To describe each drug molecule, 15 basic molecular descriptors were selected and are shown in Table S2, which are subdivided into constitutional, topological descriptors and molecular properties. These molecular descriptors of the drugs were calculated with PaDEL.^10^ The correlations between molecular descriptors and the properties of nanogels were determined with linear regression using JMP 15, with the correlation coefficient (*R*^2^) aimed above 0.7. For nonlinear correlations, the data set was fitted to compute the regression coefficients (*R*^2^) and *p*-values.

## 3 Results and Discussion

### 3.1 Central Composite Design

#### 3.1.1 Statistical Analysis

One-way analysis of variation (ANOVA) and lack-of-fit test were performed on the response surface models for each individual dependent variable to determine the statistical significance and the goodness of fit of these models on the training set, respectively. The null hypothesis of the ANOVA is that these models have no correlation to the training data set and thus do not have the predictive capacity. The results of the ANOVA and lack-of-fit tests are reported in Table 3. The *p*-values obtained in the ANOVA test for all of the models were smaller than 0.05, demonstrating the significance of the correlations between the training set and the models. Furthermore, the *p*-values in the lack-of-fit tests for all models were larger than 0.05, which indicate that these models were a good fit for the training set data. Thus, these models can predict the properties of nanogels.

**Table 3:** ANOVA and Lack-of-Fit Test Results for the CCD Models for Various Independent Variables^a^

independent variables	source of variations	degree of freedom	sum of squared	mean squares	*F* value	prob. > *F*	significance
size	model	4	46 804.498	11 701.200	649.0404	<0.001	significant
	CC	1	46 444.225		2576.256	<0.0001	significant
	CT	1	0.529		0.529	0.0293	not significant
	CC × CT	1	182.405		10.118	0.0079	significant
	CT^2^	1	177.738		9.859	0.0085	significant
	pesidual	12	216.342	18.000			
	lack of fit	4	49.794	12.449	0.5980	0.6746	not significant
	pure error	8	166.548	20.819			
PDI	model	3	0.117	0.039	56.7929	<0.001	significant
	CC	1	0.061		88.579	<0.001	significant
	CT	1	0.055		79.523	<0.001	significant
	CC × CT	1			2.277	0.1552	not significant
	residual	13	0.009	0.001			
	lack of fit	5	0.006	0.001	3.2981	0.0654	not significant
	pure error	8	0.003	0.000			
EE	model	2	0.032	0.0162	3.9079	0.0448	significant
	CC	1	0.022		5.347	0.0365	significant
	CT	1	0.010		2.468	0.1385	not significant
	residual	14	0.058	0.004			
	lack of fit	6	0.003	0.000	0.0686	0.9979	not significant
	pure error	8	0.055	0.007			
ZP	model	6	251.138	41.856	13.2271	0.00003	significant
	CC	1	66.641		21.060	0.0010	significant
	CT	1	50.060		15.820	0.0026	significant
	CP	1	19.721		6.232	0.0316	significant
	CC × CT	1	22.616		7.147	0.0234	significant
	CC × CP	1	26.082		8.242	0.0166	significant
	CC^2^	1	66.017		20.862	0.0010	significant
	residual	10	31.644	3.164			
	lack of fit	8	28.035	3.504	1.9420	0.3839	not significant
	pure error	10	3.609	1.805			

a Where CC is chitosan concentration, CT is the chitosan/TPP ratio, and CP is the chitosan/propranolol ratio.

#### 3.1.2 Effect of Factors on the Nanogels

##### 3.1.2.1 *Z*-Average and Polydispersity

Size is one of the important factors controlling the performance of the nanogels in cellular uptake.^5^*Z*-average is measuring the hydrodynamic size of the nanoparticles, which is a better indication of the size of the nanogels in solution than the size measured in TEM as the latter measures the dried state. All nanogels in the 17 formulations from the training set were found to be within a range from 56.8 to 208.1 nm. These nanoparticles were in the range for endocytic uptakes. Chitosan concentration, the interaction effect between the chitosan concentration and chitosan/TPP ratio, and the quadratic effect of the chitosan/TPP ratio were found to have significant effects on the *Z*-average of nanogels. Although the effect of the individual term for the chitosan/TPP ratio was not significant, it had to be included in the model as its interaction and quadratic terms were included.

**Figure 1:**
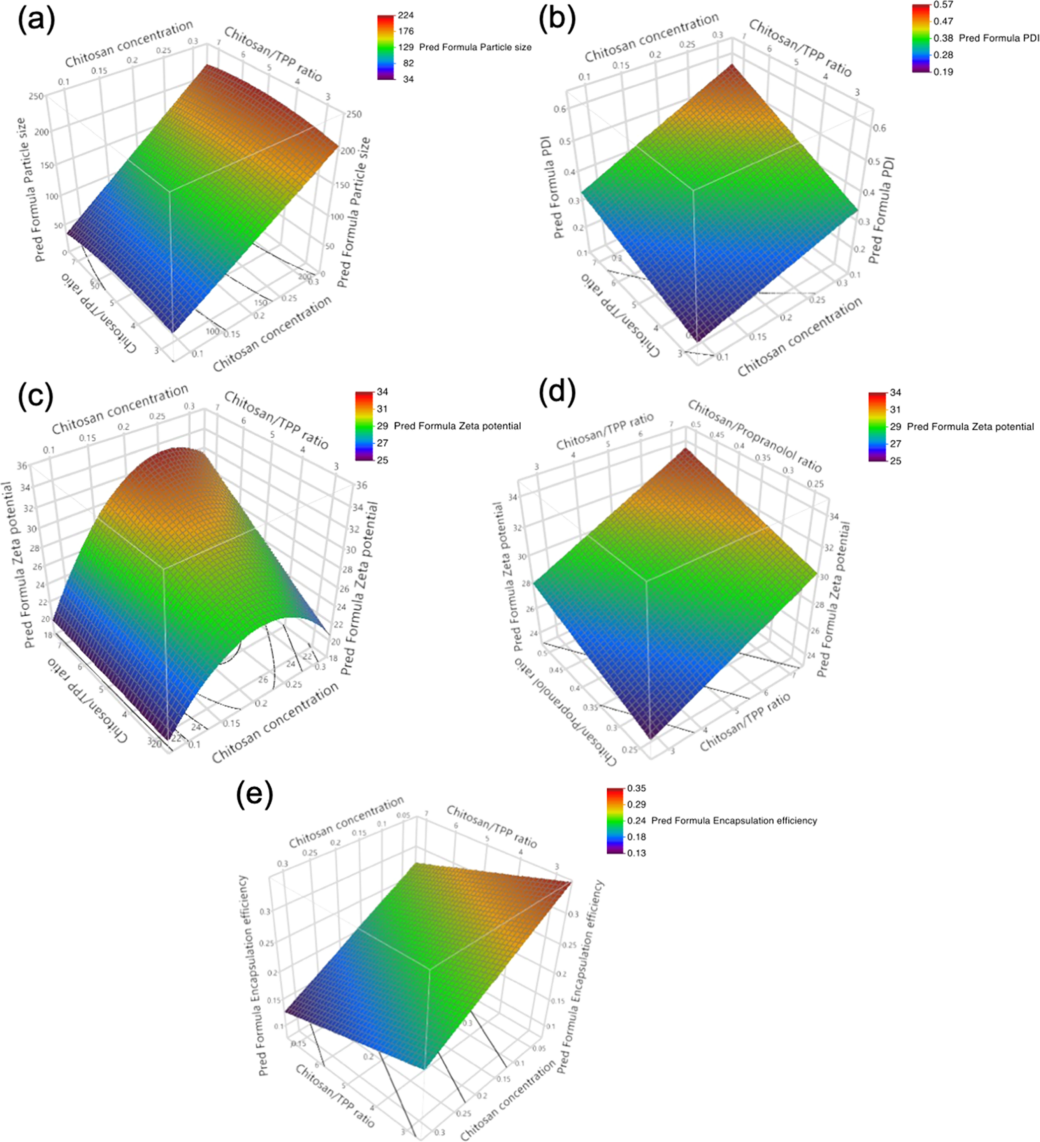
Response surface models predicting the effect of chitosan concentration and chitosan/TPP ratio on (a) *Z*-average, (b) PDI, and (e) %EE. As the response surface and contour plots are only able to compare two factors at once, the model of ZP was presented in panels (c) and (d), showing the effect of chitosan concentration against chitosan/TPP ratio and chitosan/TPP ratio against chitosan/propranolol ratio, respectively, on ZP.

The size of the nanogels increased with the chitosan concentration, as shown in Figure 1a. However, the observed trend is opposite to the results reported by Al-Kassas et al.,^15^ in which the “one-factor-at-a-time” (OFAT) optimization approach was used to prepare propranolol-loaded chitosan–TPP nanogels. Nanogels fabricated in their study with a 0.1% chitosan were much larger than those prepared from 0.2 and 0.3% chitosan. Moreover, the size of the nanogels prepared in this study was generally smaller than those reported by Al-Kassas’ group. The discrepancy in nanogel sizes between the two studies is probably due to a different grade of chitosan being used, with low-molecular-weight chitosan used in this study while medium-molecular-weight chitosan being used by Al-Kassas et al. As chitosan concentration increases with the viscosity, the cross-linking between chitosan and TPP is inefficient at high chitosan, eventually forming larger particles.^18^ Furthermore, an interaction between chitosan concentration and the chitosan–TPP ratio was identified in the response surface model, which demonstrated that the nanogels formed were bigger at high chitosan concentration and high chitosan–TPP ratio. It is likely due to more chitosan and TPP being available and thus more cross-linking was formed
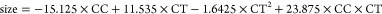
3On the other hand, polydispersity is a less crucial factor in endocytosis in contrast to the size, as endocytosis is still feasible for chitosan–TPP nanogels even though a wide range of nanoparticles with different sizes were present.^19,20^ Ma et al. successfully delivered large (>400 nm) and polydisperse (PDI = 0.5) chitosan–TPP nanoparticles to small intestine Caco2 cell lines.^20^ However, from a pharmaceutical perspective, a successful nanoformulation should be stable, safe, and effective, and the preparation method should be robust. Thus, the population of the nanocarriers should be as homogeneous as possible. The PDI is a measure of the homogeneity of the nanoparticles in terms of size distribution,^21^ which is a value between 0 and 1 for the Malvern Zetasizer series. Hence, the smaller the PDI, the more uniform the size of the nanogels. A high PDI value (>0.7) denotes a very broad size distribution of the nanoparticles, which might indicate agglomeration of the nanoparticles or the presence of other contaminants.

PDI of the training set formulations was between 0.217 and 0.507, of which only four among these formulations were considered polydisperse (PDI > 0.4) and the rest of the formulations were moderately dispersed. The response surface model is shown in Figure 1b, where chitosan concentration and chitosan/TPP ratio were identified to have a significant effect on the PDI of the nanogel formulations. The findings are similar to the trend observed in the study performed by Hosseinzadeh et al.^22^ It is likely due to more cross-linking formed at high chitosan concentration and high TPP concentration (i.e., chitosan/TPP ratio), forming larger particles and agglomerating. Therefore, with larger particles being formed, the distribution of size for the nanogels was broadened and higher PDI was observed. However, their interaction was not significant, albeit it was estimated in the stepwise regression, which indicated that the nanogels are more polydisperse at high chitosan concentration and chitosan/TPP ratio, with no synergetic effect observed between these two factors
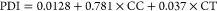
4

##### 3.1.2.2 ζ Potential

The nanogels are formed by ionic gelation between cationic chitosan and anionic TPP, where chitosan is generally used in excess compared to TPP. Therefore, nanogels are generally positively charged at acidic conditions with pH < 6, where the amine group on the chitosan is protonated. The ZP of nanogels is an important influencer on the colloidal stability of the nanogels, as the agglomeration of nanogels is attenuated by the electronic repulsion.^22,23^ Nanogels with ZP values of 30 mV are generally stable in suspension due to the sufficient electronic repulsion between particles.^24^ ζ potentials of the nanogels from the training set, as shown in Table 1, were in a range of 18–32 mV, which indicated that only a quarter of the nanogel formulations were stable due to the surface charge in the suspension.

All selected factors were found to have significant effects on the ZP of the nanogels. Positive coefficients were associated with the chitosan concentration and chitosan/propranolol ratio in eq 5, which indicated that the ZP of nanogels increased with these parameters. As chitosan and propranolol consist of amine groups and are positively charged at pH 4.5, increasing chitosan and propranolol concentration will result in more positive charges on the nanogel particles. At high chitosan concentration, the cross-linking between chitosan and TPP is ineffective and thus the ZP is higher at high concentration.^18^ In contrast, TPP is an anionic molecule, of which increases in chitosan/TPP ratio will lead to a decreasing amount of TPP for cross-linking and reduction of the negative charge on nanogels; thus, the ZP is inversely correlated to the chitosan/TPP ratio. These findings were in good agreement with the study conducted by Al-Kassas et al., using the OFAT approach.^15^ Design of experiment approach is generally more advanced and allows interactions and quadratic effects to be identified, compared to the OFAT approach. Therefore, several additional factors were identified influencing the ζ potential of the nanogels in the RSM model. A quadratic effect of the chitosan concentration is demonstrated in Figure 1c,d, which illustrates that there is a maximum concentration for chitosan at 0.25% to achieve the highest ζ potential. Interactions between chitosan concentration and chitosan/TPP ratio, as well as between chitosan concentration and chitosan/propranolol ratio, were identified in the model, which demonstrated the relationships among these factors, and the effect of chitosan concentration on the ζ potential is dependent on the other two factors
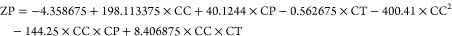
5

##### 3.1.2.3 Encapsulation Efficiency

Another crucial property of nanogels is their ability to encapsulate therapeutic molecules. The %EE of propranolol in the nanogel formulation was between 10 and 40%, as shown in Table 1, which indicates that the encapsulation process of propranolol was inefficient. On the contrary, Al-Kassas et al. reported that the %EE in their study was over 85%.^15^ The discrepancy is probably related to the nanogels formed using low-molecular-weight chitosan in this study. Chitosan concentration and chitosan/TPP ratio were found to have inverse effects on the %EE, with low %EE observed at high chitosan concentration and low chitosan/TPP ratio (i.e., high TPP concentration), as shown in Figure 1e. It is likely due to the inefficient cross-linking at these conditions. Moreover, Whiteley et al. used a similar central composite design to predict the encapsulation efficiency of lysozyme in nanogels fabricated via microfluidics.^25^ The %EE of lysozyme in nanogels was higher, with at least 54% of the drug input. One possible reason to account for the discrepancy in encapsulation efficiency is that the molecular weight of propranolol and lysozyme is massively different of 259.34 and 14.3 kDa, respectively, even though both are carrying a positive charge. Propranolol is, therefore, more likely to leach out from the nanogels, as compared to lysozyme. Moreover, the study identified different important factors on %EE, which also revealed that the encapsulations of payloads are different between microfluidics and stirring
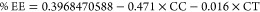
6

#### 3.1.3 Multiple Response Optimization

The optimal fabricating condition was determined by multiple response optimization (MRO), as shown in Figure S2, aiming to achieve the highest %EE and ZP and the lowest Z-average and PDI. The optimal running condition for nanogel production is at 0.10% chitosan concentration, a chitosan–TPP mass ratio of 3, and a chitosan–propranolol mass ratio of 0.5, as shown in Table 4. The predicted size, PDI, ZP, and %EE of nanogels produced at the optimal condition were 69.2 nm, 0.217, 25.3 mV, and 30.1%, respectively, while the measured results of the nanogels were 75.5 nm, 0.210, 31.4 mV, and 66.0%. The measured values were 9.1, 2.8, 24.1, and 122.3% different from the predicted values, respectively. The high discrepancies between the measured and predicted values for %EE demonstrate that the model did not give a good prediction and was dependent on the training set.

**Table 4:** Desired Formulations of Propranolol-Loaded Chitosan Nanogels, with the Parameter, Investigated, Experimental Findings, and Predicted Results

	experiment conditions			size (nm)			PDI			ZP (mV)	EE (%)
sample	CC (%)	CT	CP	exp	pred	%diff	exp	pred	%diff	exp	exp
F1 (opt.)	0.1	3	0.5	75.5 ± 2.2	68.9	–8.7	0.210 ± 0.013	0.211	0.5	31.36 ± 1.34	66.0 ± 0.9
F2	0.15	3	0.5	97.0 ± 1.5	102.3	5.5	0.247 ± 0.009	0.231	–6.5	34.26 ± 1.42	69.0 ± 6.5
F3	0.3	5	0.5	186.8 ± 2.0	200.1	7.1	0.461 ± 0.009	0.385	–16.5	40.85 ± 1.40	66.1 ± 6.2

#### 3.1.4 Test Sets and Final Formulations

**Figure 2:**
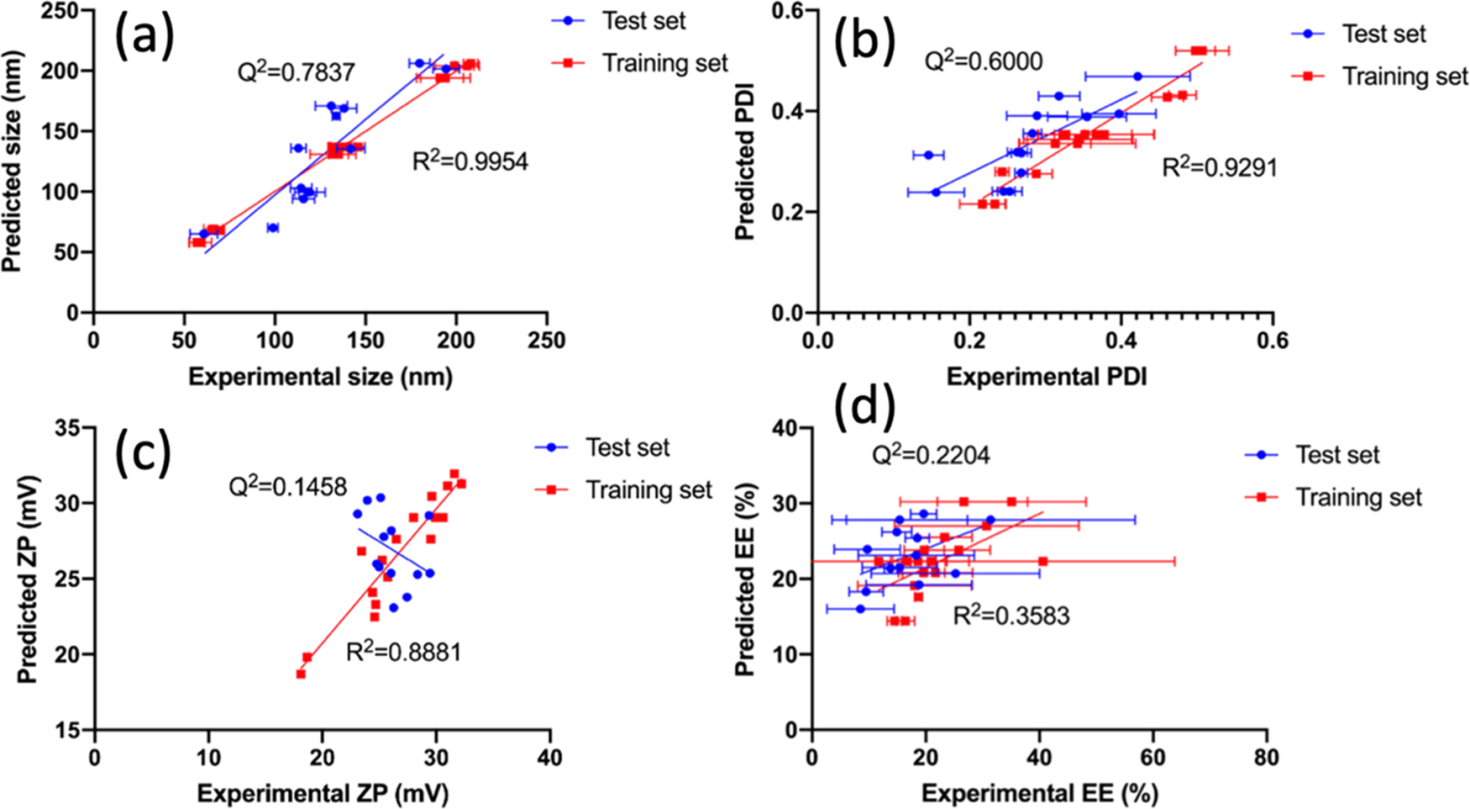
Correlations between measured and predicted values for the prediction of (a) size, (b) polydispersity (PDI), (c) ζ potential (ZP), and (d) encapsulation efficiency (EE) for the training and test set formulations.

Thirteen test set formulations were performed to determine the prediction accuracy of the models, with the measured and predicted results shown in Table 2. The regression coefficients of the test set (*Q*^2^) were compared to those of the training set (*R*^2^) for each parameter of nanogels. Good fitting is reflected on *R*^2^ closer to 1, while similar *R*^2^ and *Q*^2^ indicate that the model was working independently from the training data set, which indicates the power of model prediction. Size and PDI models were good with the relatively high *Q*^2^ value (>0.6) compared to *R*^2^, as shown in Figure 2, indicating high predictive accuracy. As these models work independently from the training data set, the model and mathematical equation could be used for prediction. Conversely, the models for ZP worked only on the training set and had limited predictive accuracies. An opposite trend is observed for the test set compared to that for the training set. Therefore, the model for ZP should not be used for prediction and as the criteria for final formulation. The *R*^2^ and *Q*^2^ for the EE model were low, which suggested that the model is not a good representation of the %EE within the design space nor having a good prediction ability. Therefore, only the hydrodynamic size and PDI of the nanogels were predicted, as shown in Table 4, while the ZP and %EE were only measured. The results elucidated the importance to verify the models with test sets as the constructed models do not necessarily have the power of prediction.

Two other formulations (F2 and F3) were produced according to the predicted experimental conditions identified in the contour plots to produce nanogels with the smallest PDI and size of 100 and 200 nm, respectively. These contour plots are displayed in Figure S4, while the identified experimental conditions are presented in Table 4. The differences between the predicted size and PDI were less than 10% in these formulations, which elucidated that these models are reliable and accurate in predicting the experimental conditions to obtain the nanogels with desirable properties, in addition to identifying the optimal fabricating conditions through the desirability function. The ζ potential and encapsulation efficiency were measured but not predicted, owing to the lack of predictability.

### 3.2 Characterizations of Raw Materials and Freeze-Dried Nanogels

#### 3.2.1 Fourier Transform Infrared Spectroscopy

**Figure 3:**
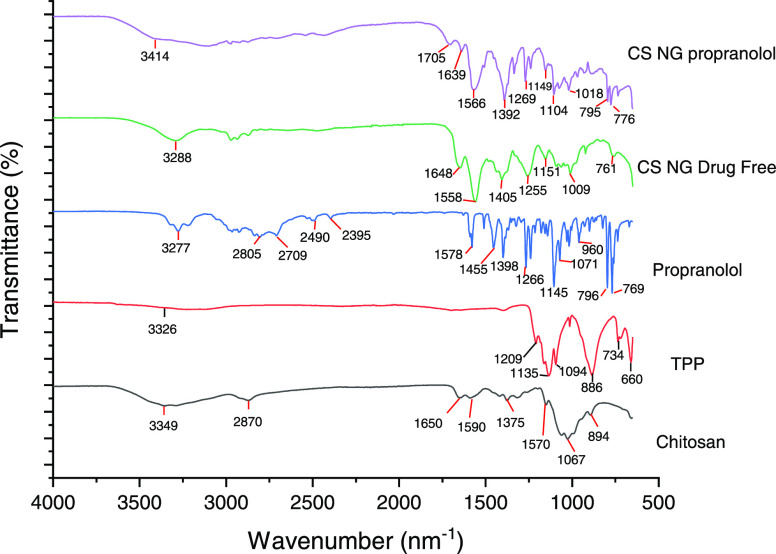
FTIR spectrum showing the components of the formulation individually, freeze-dried unloaded nanogels, and the optimum formulation of propranolol-loaded nanogels.

Figure 3 shows the IR spectrum of the individual components of the nanogels, as well as both propranolol-loaded and drug-free nanogels. In the spectra of LMW chitosan, strong bands around 3290 and 3356 cm^–1^ are associated with O–H and N–H stretching and intramolecular hydrogen bonds, while the peak at around 2870 cm^–1^ corresponds to asymmetric C–H stretching. Similar bands at 3288 and 3414 cm^–1^ were observed in freeze-dried nanogels, which also correspond to these intramolecular hydrogen bonds. The symmetric C–H stretching was not obvious in the spectra, as no peak was observed around 2900 cm^–1^. N-Acetylation of chitosan was confirmed by the bands at 1642–1650 and 1323 cm^–1^, which are the C=O stretching and C–N stretching of amide, respectively, as well as the peak at around 1590 cm^–1^, which corresponds to the N–H bending. The strong bands at 1027–1068 cm^–1^ are associated with the C–O stretching. The spectra agree with the result reported in the literature.^26,27^ In the IR spectrum of propranolol, a band at 3277 and 3221 cm^–1^ corresponds to the O–H and N–H stretching with intramolecular hydrogen bonds, respectively. A peak at 796 cm^–1^ corresponds to the naphthalene in propranolol, while the aryl alkyl ether is associated with the peak at 1266 cm^–1^.^28^ C=C stretching in naphthalene is observed with a sharp peak at 1578 cm^–1^. The spectrum obtained agrees with other studies.^29^ As for TPP, the band at 3326 cm^–1^ corresponds to the O–H stretching, while the band at 1135 and 1209 cm^–1^ associates with O–P=O and P=O stretching, respectively.^30^ A sharp peak at 1094 cm^–1^ corresponds to the P–O stretching. The sharp peaks at 1255 and 1269 cm^–1^ were present in the drug-free and propranolol-loaded nanogels, respectively, which are indicative of the P=O bond in TPP within the nanogel structure, albeit shifted from 1209 cm^–1^ in TPP alone as a result of the interaction with chitosan.^25^ Drug-free nanogels exhibited sharper peaks at 1558 and 1648 cm^–1^ compared to the chitosan, which showed that the complexation of chitosan with TPP is likely to influence the chemical interaction between chitosan. Moreover, the C–O stretching of either group in chitosan was observed at 1087 cm^–1^, which shifted to 1009 and 1018 cm^–1^ in the drug-free and propranolol-loaded nanogels. The shift was similar to the reported literature.^25^ Moreover, several distinct peaks for propranolol at 776, 795, and 1269 cm^–1^ were present in propranolol-loaded nanogels, which were not observed in the drug-free nanogels. In conclusion, the IR spectrum confirms the presence of the individual components in the nanogels and structural change of the nanogels after encapsulation and loading of propranolol was not observed.

### 3.3 Drug Release Study

**Figure 4:**
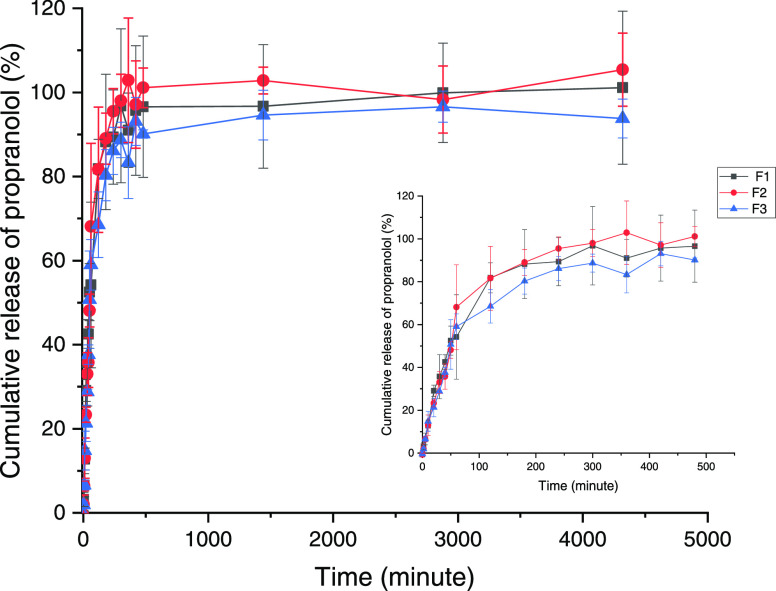
In vitro propranolol release over 72 h (large) and the zoom-in of the first 8 h (small). Data are obtained from three independent experiments and represented as mean ± SD.

The in vitro drug release test predicts the dissolution profile and bioavailability of the loaded drugs. The use of PBS (pH 7.4) is to stimulate the release at physiological pH. A burst release of encapsulated propranolol in the first 2 h can be observed in Figure 4. A significant release of around 20% propranolol was observed within the first 10 min, followed by a slow increase until 8 h, where nearly all of the drugs were released from the carrier. After 8 h, the concentrations from some formulations leveled off. Similar release profiles were observed between nanogels F1–F3, which indicated that the nanogels are likely to release propranolol in a similar way regardless of the size and PDI of the nanogels were different. It is likely due to the precipitation and aggregation of chitosan nanogels in PBS. The solutions turned turbid and opaque due to the presence of participates. Precipitation of chitosan might also destroy the architecture of nanogels, and thus propranolol inside the void of nanogels might leach out, which might account for the rapid and burst release. Besides, as both propranolol and chitosan are cationic, there is lacking interaction between polymer and propranolol to retain the propranolol and slow the release down. The number of precipitates present in the dialysis bag was likely to be dependent on the concentration of chitosan and therefore the amount of chitosan present, with the lowest amount of precipitate observed in F1 and the highest amount of precipitate observed in F3 for LMW chitosan.

### 3.4 Application of the Prediction Models to Other Drugs

**Figure 5:**
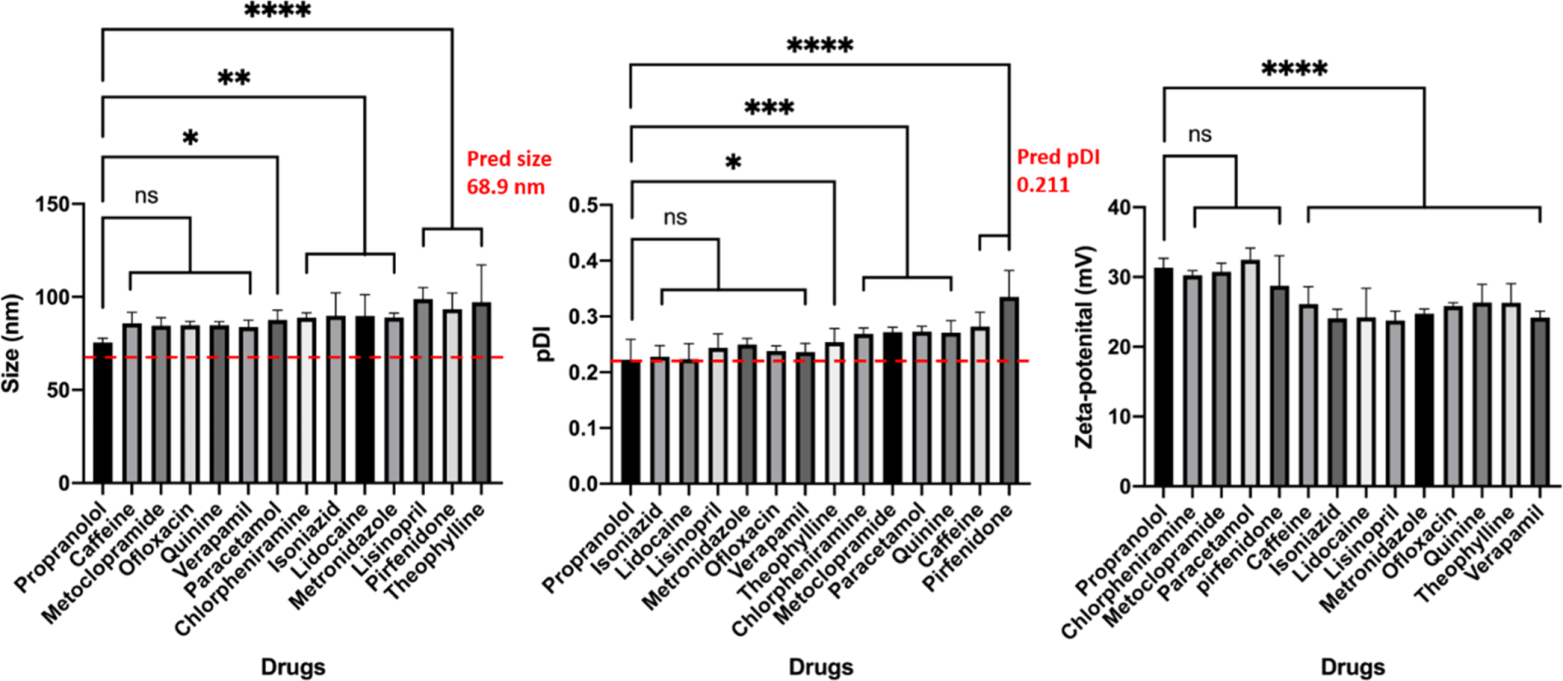
Measured size, PDI, and ζ potential of the nanogels fabricated with a variety of drugs with no structural similarity with propranolol. The red dashed lines represented the predicted value from the model. Analysis of variance (ANOVA) was performed to determine the significant difference between nanogels loaded with propranolol and other drugs. The error bar represents the standard derivation. *p-Value <0.05, **p-value <0.01, ***p-value <0.001, and ****p-value <0.0001. ns refers to a *p*-value >0.05. All samples were fabricated at the optimal conditions, with chitosan concentration at 0.1% (w/v) and chitosan/TPP ratio and chitosan/propranolol ratio of 3 and 0.5, respectively.

Nanogels were fabricated at the optimal condition identified in the model using a total of 19 other drugs, where the drugs are divided into two groups based on the molecular similarity to propranolol. The measured size, PDI, and ζ potential of the nanogels fabricated using structurally distinct drugs with propranolol are shown in Figure 5. Dunnett’s test was applied to the measured size, PDI, and ζ potential of the nanogels fabricated with different drugs, which illustrated that the properties of the nanogels were different and were dependent on the choice of drugs. Moreover, the percentage differences between the predicted and measured sizes of nanogels were ranged between 10 and 41%, while the counterpart in PDI was between 5 and 59%. The discrepancy between the predicted and measured values elucidated that the model does not apply to other drugs, and DOE optimization should be performed when a different drug is used. These drugs are structurally distinct from each other (i.e., no structural–activity relationship (SAR)), possess different functional groups, and thus are likely to interact with chitosan and TPP in nanogels differently, influencing the properties of the nanogels. Albeit the distinct results from the propranolol-loaded nanogels, all other nanogels were between 70 and 120 nm in size and the PDI were between 0.2 and 0.4. The ζ potential of the nanogels was above 20 mV, which indicated that the nanogels were less stable than the propranolol-loaded nanogels but possessed some degree of colloidal stability. The results indicated that the size, PDI, and ZP were different but remained in similar magnitudes, indicating that properties of nanogels are partly dependent on the formulation.

To investigate whether drugs with molecular similarities behaved differently, seven β-blocker drugs with structure–activity relationships (SARs), including propranolol, were tested. All β-blocker drugs had similar structures, the same number of acid and base groups, and p*K*_a_ of ∼pH = 9. Thus, at pH 4.5, the drugs were ionized and likely interacted with anionic TPP in the nanogels. However, there are also repulsions between cationic chitosan and drugs, which could limit the encapsulation and decrease the drug loading. As the drugs and TPP are polar and possess a hydrogen acceptor and donor, these interactions are likely to play a role in drug loading. Multiple linear regressions and nonlinear fittings were conducted to evaluate the correlations between the selected molecular descriptors and nanogel properties, which are shown in Table 5. Metoprolol was not included in the fittings, as the drug comes as a tartrate salt of which tartrate could also cross-link in chitosan nanogels.^31^ Thus, the extra cross-linking may mask the effect of the drug itself. As the sample sizes were too small with only seven β-blockers, these drugs were not subgrouped into training and test sets.

**Table 5:**
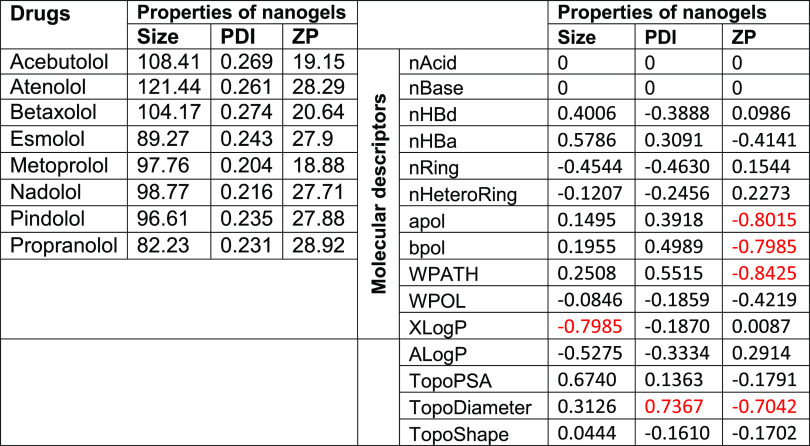
Estimated Correlation Coefficients (*R*^2^) between Selected Molecular Descriptors and Properties of Nanogels Were Calculated by the Row-Wise Method^a^

a *R*^2^ < 0.5 referred to weak effect, 0.5 < *R*^2^ < 0.7 indicated moderate effects, while *R*^2^ >0.7 indicated strong correlation. The estimated correlations with *R*^2^ >0.7 were highlighted in red. A negative value in *R*^2^ indicated an inverse correlation and vice versa.

**Figure 6:**
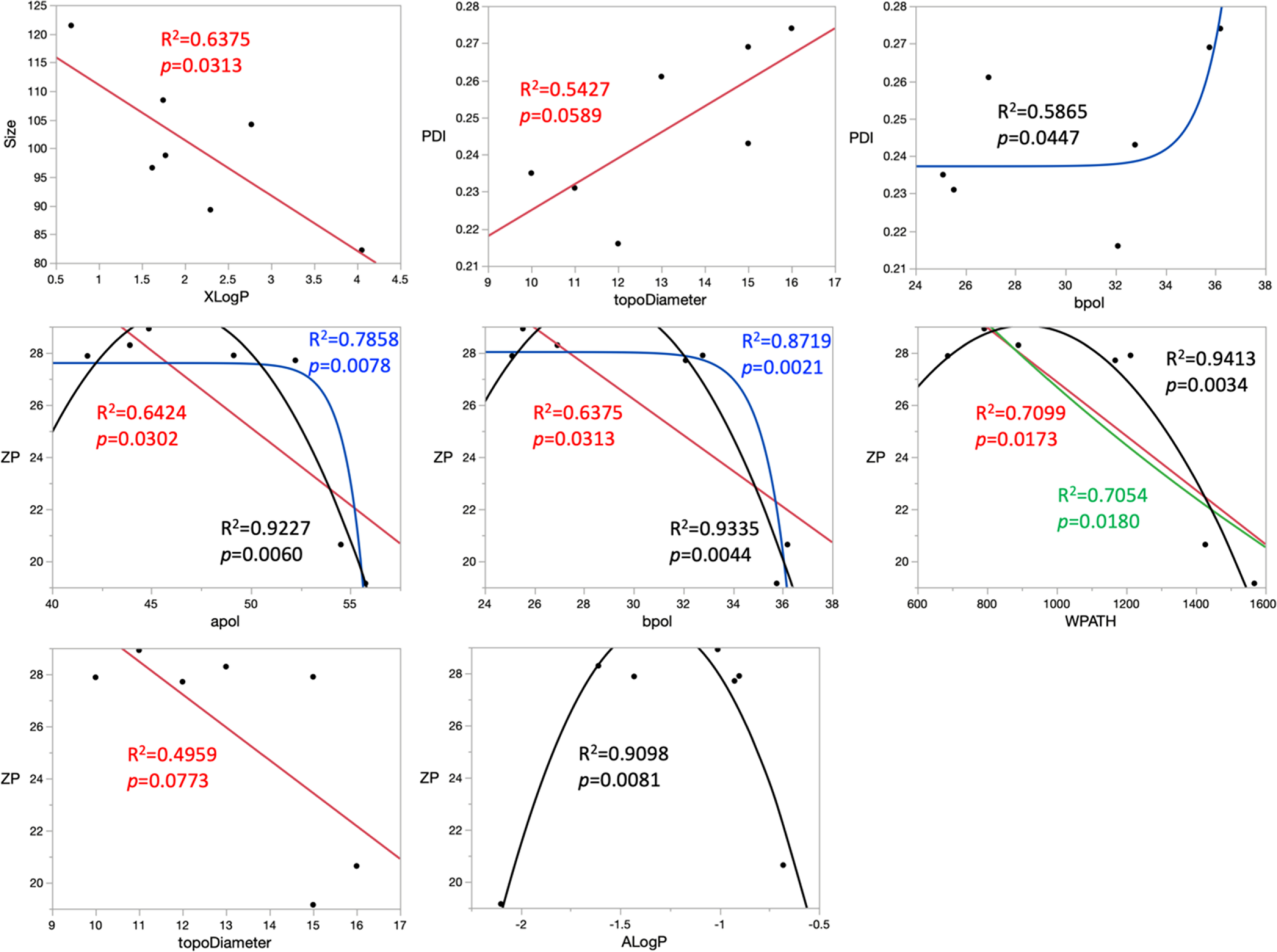
Linear regression and nonlinear correlation plots between the nanogel properties and the selected molecular descriptors. Linear, logarithmic, exponential, and quadratic fittings were plotted in red, green, blue, and black, respectively, with the correlation coefficient (*R*^2^) and *p*-value for each fitting shown in the respective color.

Several strong linear correlations were estimated by the row-wise method between some molecular descriptors and properties of nanogels. As the number of acid and base in all tested β-blockers are the same, the correlation coefficients were 0. Correlations with strong effects were estimated between XLog *P* and size, as shown in Figure 6. Both ALog *P* and XLog *P* are atom-additive approaches to calculate the partition coefficient (Log *P*) theoretically, where XLog *P* is an enhanced modification of ALog *P*. The correlation coefficient (*R*^2^) between the size and XLog *P* was 0.64, and the *p*-value was 0.0313, which demonstrated that the correlations were moderately strong and statistically significant. Hence, the nanogels are likely to be smaller in size when a β-blocker with higher XLog *P* is used. As Log *P* is a measure of hydrophobicity, higher XLog *P* indicates higher hydrophobicity. Therefore, the observation elucidates that the nanogel size reduced with the hydrophobicity of β-blockers. Another strong correlation was estimated between PDI and topological diameter (TopoDiameter), indicating a potentially strong influence of graph-theoretical sizes on the PDI of the nanogel as TopoDiameter is a measure of the maximum atom eccentricity. However, the *p*-values for the ANOVA and lack of fit were 0.0589 and 0.7043, respectively, which indicated that the relationship was well fitted but was statistically insignificant. Other multiple correlations were estimated between the sum of all atomic polarizability (apol), the sum of the absolute value of the difference between atomic polarizabilities of all bonded atoms in the molecule (bpol), Wiener path number (WPATH), and TopoDiameter with ζ potentials. Apol and bpol are two measures of the polarizability of the drug, while WPATH is a topological descriptor, which is defined as the sum of the lengths of the shortest paths between all pairs of vertices in the chemical graphs.^32^ The Wiener index helps to identify the branching, cyclicity, and centricity of the compounds. The first three correlations were statistically significant, with a *p*-value of 0.0302, 0.0313, and 0.0173, whereas the correlation between TopoDiameter and ZP was not. All regression coefficients were over 0.6, which demonstrated that these correlations were moderately strong. The result revealed that the polarizability and molecular topography of the drug could potentially affect the ζ potential of the nanogels.

Nonlinear correlations, such as logarithmic, exponential, and quadratic correlations, were also fitted between the molecular descriptors and the nanogel properties. As with the linear regressions, the correlation coefficients for nAcid and nBase were 0. Interestingly, the exponential correlation between PDI and bpol was significant despite the moderate effect of the correlation, with a *p*-value of 0.0447 and an *R*^2^ of 0.5865. The result revealed that the polarizability of the drugs impacted the PDI of the nanogels, potentially in an exponential growth fashion. Moreover, the interactions between apol, bpol, WPATH, and ZP could be fitted in other relationships, as shown in Table 6, with the quadratic correlations between these molecule descriptors and ZP deemed as the best fitting (*R*^2^ > 0.9). However, the vertex of the quadratic fit could not be confirmed by the existing data set and thus is likely to be overfitted. Exponential fitting between apol and ZP, as well as bpol and ZP, was also good, with *p*-values of 0.0078 and 0.0021, respectively. Hence, the result showed that these two factors could have negative exponential effects on the ZP, instead of linear relationships. Contrarily, the exponential fit was invalid for WPATH and ZP, as there are invalid arguments. The quadratic fit was the best fit among all relationships, with the highest *R*^2^. Both linear and logarithmic correlations were comparable and slightly above 0.7. With the existing data set, there is likely a quadratic correlation between the WPATH and ZP. Last but not least, a new correlation between ALog *P* and ZP was identified in the quadratic fit, with a *p*-value of 0.0081 and an *R*^2^ of 0.9098. The result indicates that hydrophobicity also influenced the ZP and the colloidal stability of the nanogels.

**Table 6:**
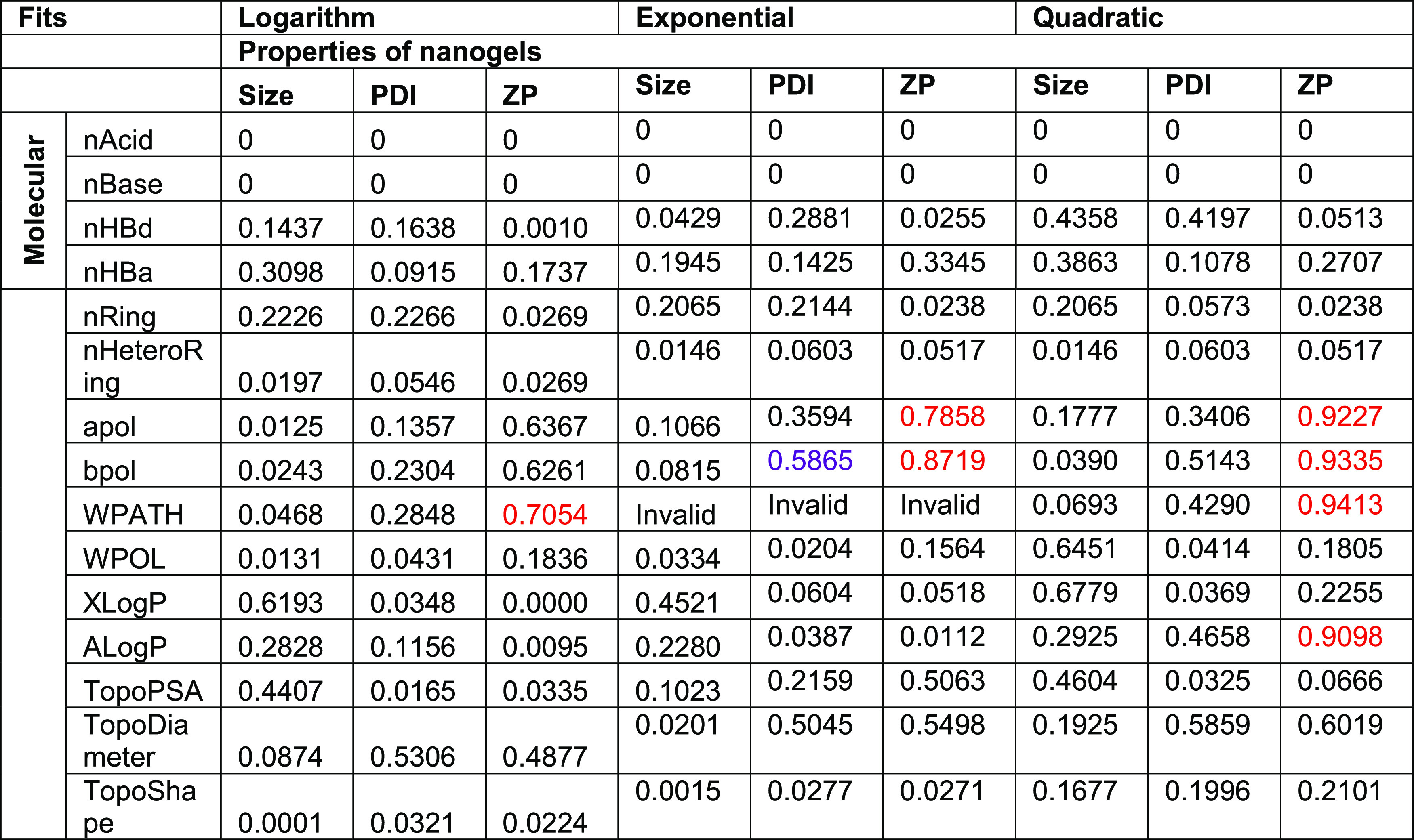
Correlation Coefficients (*R*^2^) between Selected Molecular Descriptors and Properties of Nanogels Were Calculated for Each Nonlinear Fitting Method^a^

a The correlations with *R*^2^ > 0.7 and *p* < 0.05 were highlighted in red, while the correlations with *R*^2^ < 0.7 and *p* < 0.05 were highlighted in purple.

To summarize, the results indicate that the properties of the payload impacted the properties of nanogels. Interaction between the drugs and other components of nanogels is most likely to play an important role in determining the nanogel properties in addition to the formulations. Thus, the loading of drugs in nanogels is not purely a simple entrapment inside the void. Drugs with various sizes and shapes could still be encapsulated in the nanogels but altered the nanogel properties. Furthermore, most of the topological size and shape descriptors selected failed to influence the nanogel properties, which also supported the finding. Interestingly, since the structural difference between drugs is small for drugs with structural activity relationships, hence the interactions between nanogel and drugs remain similar. The constitutional descriptors selected did not impact the nanogel properties, which demonstrated that the hydrogen bondings and the heterocyclic and aromatic rings were not the key interactions or groups between the drugs and the carrier. Instead, the hydrophilicity, polarity, and polarizability of the drugs were more important, which were found to impact the nanogel properties. With these correlations, the DOE models established could potentially apply to similar drugs to estimate the nanogel properties. In practice, for example, if the target payload is expensive or has limited availability, cheaper drugs with an SAR could be used to optimize the formulation and the optimum conditions could then be applied to the target payload.

As a proof-of-concept study, there are several limitations to this approach. First, only a very small number of molecular descriptors were selected compared to approximately 1800 descriptors computed by PaDEL. Therefore, the future use of machine learning could help identify the molecular descriptors that have stronger and more nonlinear correlations with the properties of nanogels, as well as from a larger pool of molecular descriptors. Despite the limitations, this study revealed that the established DOE models could be applied to similar drugs with the help of molecular descriptors. It also provided a deeper understanding of how drugs are loaded in nanogels as well as how payloads could impact the properties of nanogels.

## 4 Conclusions

Prediction models for the properties of propranolol-loaded nanogels were constructed using a DOE approach. Three investigated factors were chitosan concentration, chitosan–TPP ratio, and chitosan–propranolol ratio, and their effects on the hydrodynamic size, PDI, ZP, and the %EE of the nanogels were determined. Following the multiple response optimization, an optimal condition of 0.1% chitosan concentration, a chitosan–TPP ratio of 3, and a chitosan–propranolol ratio of 0.5 was predicted. The *Z*-average and PDI of the optimum nanogel formulation were 75.5 ± 2.2 nm and 0.211, respectively, which were similar to the predicted values. However, ZP and the %EE were not predicted as the predictability of these models was weak, indicating the importance of performing a test set. To evaluate the application of these prediction models to different drugs, the nanogels loaded with other drugs were fabricated at the optimal condition in the model with 12 structurally distinct and 6 structurally similar drugs, and the size, PDI, and ZP of the nanogels were measured. These properties were distinct from the predicted value, which indicated that the DOE models must be refined when a new drug is used. Nevertheless, relationships were found between structurally related drugs and performance parameters; hence, there is a dependence on the molecular structure, which could potentially be solved for a wider range of drugs. These outcomes also indicate that encapsulation and formation processes are indeed drug-dependent and are not simply a matter of incorporation into interchain voids. We therefore suggest that the interactions between the nanogels and drugs are important mechanisms for encapsulation, which also govern the properties of nanogels.

## Supporting Information Available

Calibration curve of propranolol hydrochloride; noncodified levels for the factors and prediction tools used in the central composite design; TEM images of the nanogels; and contour plots showing the conditions to fabricate F2 and F3 formulations (PDF)
