# Supplementary material for: Design of Experiment Approach to Modeling the Effects of Formulation and Drug Loading on the Structure and Properties of Therapeutic Nanogels
Source: Mol Pharm. 2022 Jan 21;19(2):602–15. doi: 10.1021/acs.molpharmaceut.1c00699 (PMC9097514; doi:10.1021/acs.molpharmaceut.1c00699)
Supplement: Supplementary file 1 — mp1c00699_si_001.pdf [file mp1c00699_si_001.pdf]

Supporting information

A Design of Experiment Approach to Modelling the Effects of Formulation and Drug Loading on the Structure and Properties of Therapeutic Nanogels

Hei Ming Kenneth Ho[1,2], Duncan Q.M Craig[1], Richard M. Day[2*]

*** Corresponding author: r.m.day@ucl.ac.uk; tel: +44 2031082183**

[1] University College London School of Pharmacy, 29-39 Brunswick Square, London WC1N 1AX, UK

[2] Centre for Precision Healthcare, UCL Division of Medicine, University College London, 5 University Street, WC1E 6JF, UK

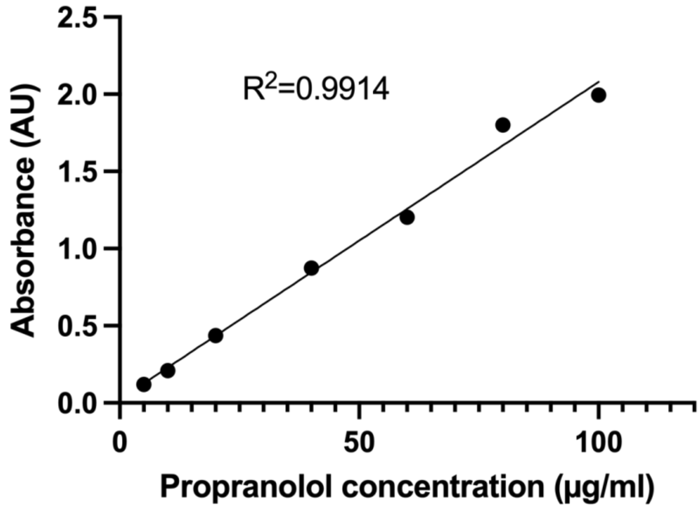

Figure S1. Calibration curve of the propranolol hydrochloride. The error bars were too small to be observed.

Table S1
Non-codified levels for each factor in the central composite design.

		Non-Codified level		
		**Low (-)**	**Mid (0)**	**High (+)**
**Factor**	**Symbol**			
Chitosan conc. (%w/v)	(CC)	0.1	0.2	0.3
Chitosan-TPP ratio	(CT)	0.010	0.015	0.020
Chitosan-propranolol ratio	(CP)	0.1	0.2	0.3

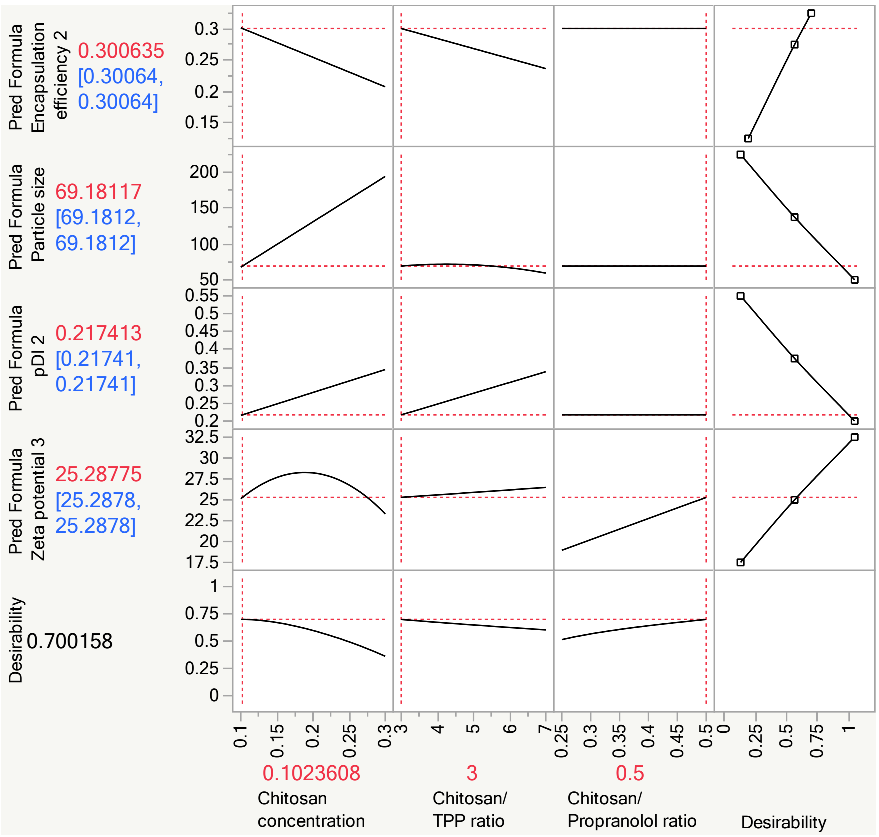

Figure S2. Prediction tool showing the relationship between the parameters and the outcome and the optimal condition for nanogel fabrications.

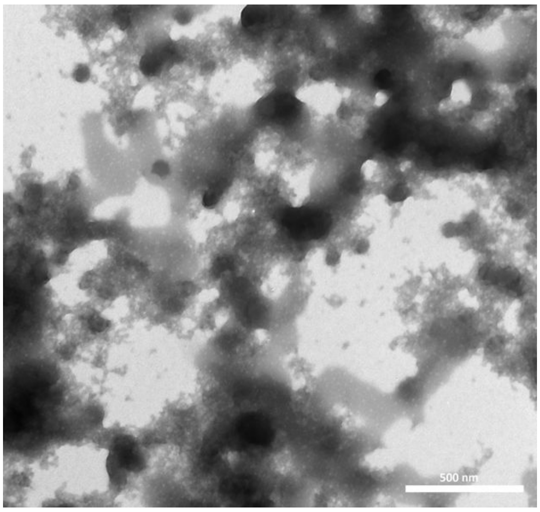

Figure S3. (Left) TEM image of the propranolol-loaded nanogels fabricated at the optimal condition

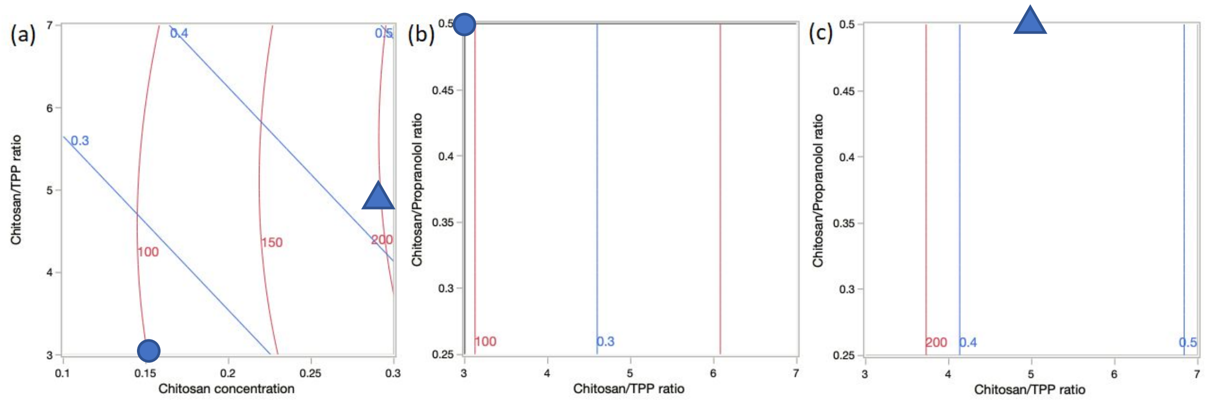

Figure S4. Contour plots of the condition to fabricate F2 and F3 formulations. (a) Contour plot of chitosan concertation against chitosan/TPP ratio, where (b) and (c) contour plots of the chitosan/TPP ratio against chitosan/ propranolol ratio at the chitosan concentrations of 0.15 and 0.3 respectively. ▯ and ▲ represented the experimental conditions for F2 and F3 respectively.

Table S2. Selected molecular descriptors

Drugs	MW	nAcid	nBase	nRing	nHBAcc	nHBDon	apol	bpol	WPath	WPol	Topo-PSA	Topo-diameter	Topo-shape	ALogP	XlogP
Propranolol	259.2	0	1	-1.0098	44.866653	25.533347	792	25	4.053	41.49	2	3	2	0	11
**Drugs without structural relationship to propranolol**															
Caffeine	194.1	0	0	-0.4311	26.75193	20.10807	258	25	-0.625	58.44	0	5	2	2	6
Chlorpheniramine	274.1	0	1	0.6514	45.209067	24.490933	718	25	4.288	16.13	0	2	2	1	9
Lidocaine	234.2	0	1	1.166	42.311446	27.648554	556	23	2.848	32.34	1	3	1	0	9
Lisinopril	405.2	2	2	-4.3984	64.940583	38.743417	2362	40	1.106	132.96	4	8	2	1	13
Metoclopramide	299.1	0	1	0.0496	46.393446	29.324554	902	29	1.144	67.59	2	6	1	0	11
Metronidazole	171.1	0	0	0.2875	22.267137	14.394863	193	15	0.017	81.19	1	3	1	1	6
Ofloxacin	361.1	1	1	-0.226	52.08086	32.83914	1484	51	1.49	73.32	1	8	4	3	12
Paracetamol	151.1	0	0	-1.0852	22.785137	11.456863	166	11	1.079	49.33	2	3	1	0	7
pirfenidone	185.1	0	0	0.1195	30.356723	14.963277	293	19	2.753	20.31	0	2	2	1	7
Quinine	324.2	0	1	-0.5136	55.007032	31.452968	1286	42	2.662	45.59	1	3	2	1	12
Theophylline	180.1	0	0	-0.967	23.658344	16.601656	211	22	-0.435	69.3	1	5	2	2	5
Verapamil	454.3	0	1	0.1993	78.266134	51.845866	3698	55	4.71	63.95	0	5	2	0	18
**Drugs with structure activity relationship with propranolol**															
Acebutolol	336.4	0	1	-2.1004	55.758204	35.761796	1568	31	1.747	87.66	3	6	1	0	15
Atenolol	266.3	0	1	-1.6098	43.915446	26.924554	890	21	0.678	84.58	3	5	1	0	13
Betaxolol	307.4	0	1	-0.68	54.522997	36.195003	1427	24	2.772	50.72	2	4	2	0	16
Esmolol	295.2	0	1	-0.9014	49.137825	32.780175	1212	24	2.296	67.79	2	5	1	0	15
Metoprolol	267.2	0	1	-0.7944	46.575825	31.822175	906	21	2.034	50.72	2	4	1	0	14
Nadolol	309.2	0	1	-0.925	52.231411	32.092589	1168	31	1.774	81.95	4	5	2	0	12
Pindolol	248.2	0	1	-1.4302	41.77986	25.10014	687	22	1.62	57.28	3	4	2	1	10
